# External jugular vein hemangioma: case report

**DOI:** 10.1590/1677-5449.180026

**Published:** 2019-04-17

**Authors:** Julio Cesar Peclat de Oliveira, Fernando Tebet Ramos Barreto, Bernardo de Castro Abi Ramia Chimelli, Lucas Maia Peclat de Oliveira, Ricardo Krapp Tavares, Thaysa Fernandes Lacerda da Costa, Diogo Di Battista de Abreu e Souza, Bianca Gutfilen

**Affiliations:** 1 Universidade Federal do Rio de Janeiro – UFRJ, Departamento de Radiologia, Rio de Janeiro, RJ, Brasil.; 2 Clínica Julio Peclat – CJP, Cirurgia Vascular, Rio de Janeiro, RJ, Brasil.; 3 Instituto Nacional do Câncer – INCA, Rio de Janeiro, RJ, Brasil.

**Keywords:** external jugular vein, neck mass, hemangioma

## Abstract

Hemangioma is a common tumor, normally diagnosed in children, and accounting for almost 10% of benign neoplasms. A hemangioma arising from the wall of a vessel is rare, and must be differentiated from other vascular malformations of the same origin. We report a rare case of a hemangioma arising from the wall of an external jugular vein and discuss diagnostic work-up and management.

## INTRODUCTION

Vascular anomalies and vascular tumors can both present as an isolated head or neck mass in adults. Their classification and subdivision into hemangiomas and vascular malformations has always been complicated and difficult to understand, since a variety of classifications and concepts have been proposed over the years, as these anomalies have been increasingly studied.^1^^-^ 3 Vascular malformations and hemangiomas that originate in the vessel wall, and particularly in the wall of the external jugular vein, are extremely rare, with fewer than 10 cases published in the literature.^4^^-^ 6 Treatment of this type of condition is primarily motivated by esthetics, since they are benign tumors.

We present the case of a patient with no prior history of vascular mass or tumors, who presented with venous ectasia in the neck, in the topography of the external jugular vein, with symptoms of pain.

## CASE DESCRIPTION

**Figure 1:**
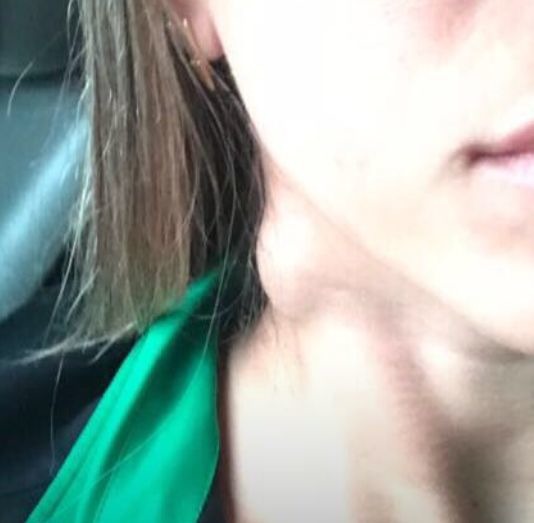
Mass in the right cervical area.

The patient was a 28-year-old female physician who engaged in regular physical activity and had no known prior diseases. She presented complaining of a mass in the right cervical region (Figure 1), causing pain when she moved her neck, and she was unable to state the time since onset of symptoms. She had no family history of vascular malformations.

**Figure 2:**
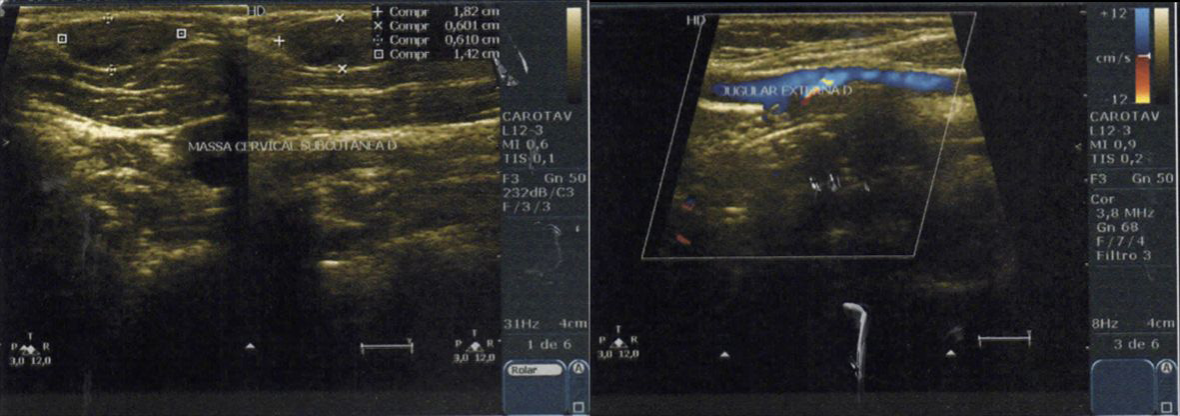
Doppler ultrasonography showing ectasia, without thrombi in the lumen and normal blood flow.

At the first consultation, she provided a Doppler ultrasonography scan of the vasculature of the neck (Figure 2), conducted on 25, November, 2016, showing the following: right external jugular vein with segmental ectasia, free from thrombi in the lumen, measuring 3.6 mm at the largest diameter. She was managed conservatively, with periodic examinations to monitor the mass. However, the patient began to present symptoms of localized pain and hardening of the mass.

On physical examination, there was swelling in the right cervical region, with hardening and mild inflammation along the path of the external jugular vein. The patient did not exhibit any other signs and her only symptom was localized pain. She was therefore medicated with rivaroxaban, for what was apparently localized thrombophlebitis, and monitored periodically with imaging exams.

**Figure 3:**
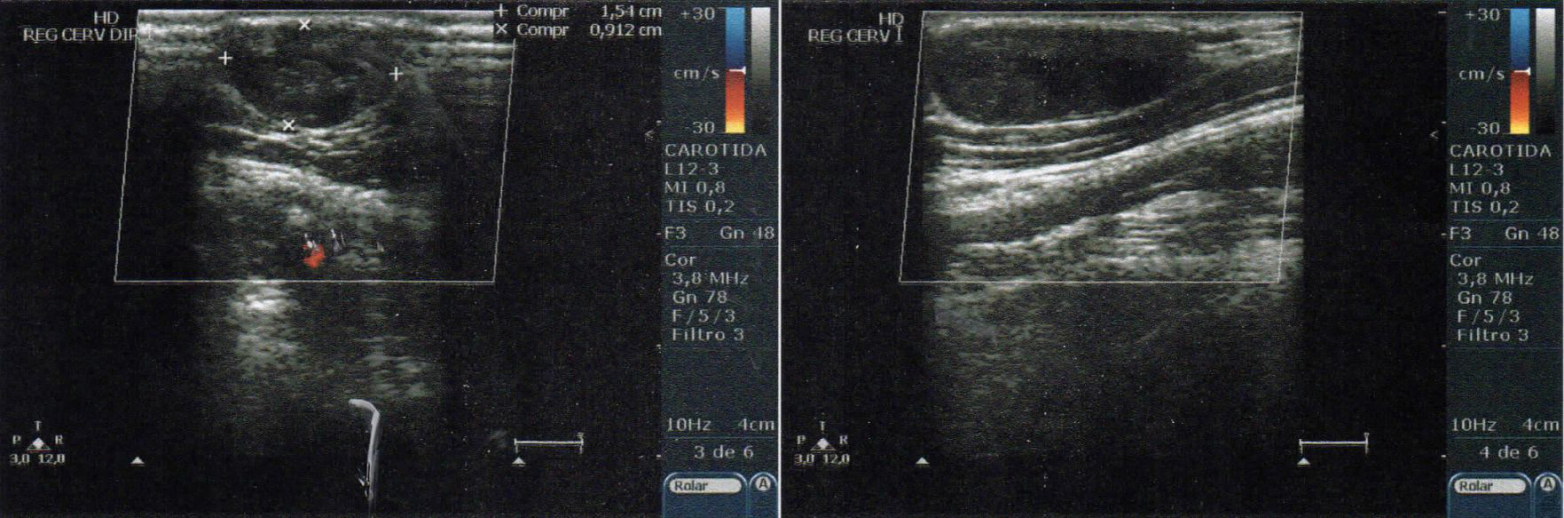
Doppler ultrasonography showing hypoechoic mass and thrombosed external jugular vein, with no flow.

After 1 year of clinical treatment, a Doppler ultrasonography scan was performed on 14 December, 2017 (Figure 3), showing a heterogeneous hypoechoic nodular formation in the upper right cervical area, measuring around 2.4 × 1.5 × 0.9 cm, in the topography of the right external jugular vein, and signs of thrombosis of the external jugular vein. The patient had exhibited little sign of improvement to the symptoms seen at the first consultation, even after clinical treatment with rivaroxaban.

**Figure 4:**
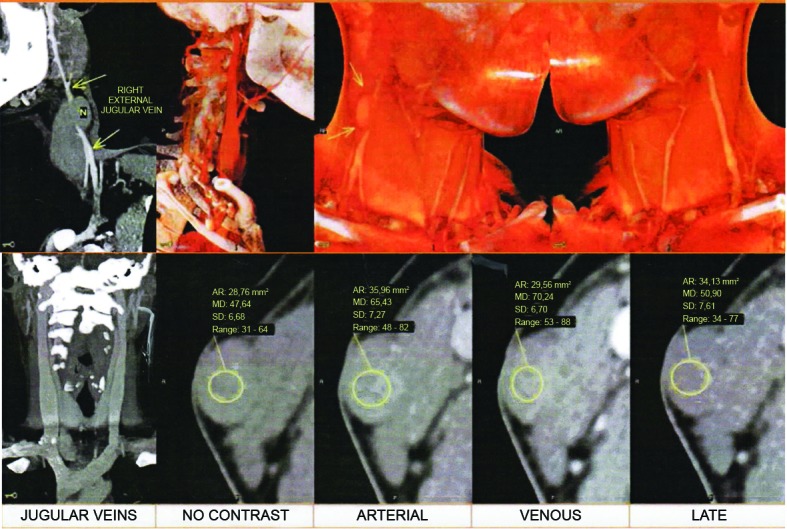
Angiotomography reconstruction demonstrating the tumoral mass and its relationship with the vascular structures of the neck. CDPI, Barra da Tijuca, RJ, Brazil. AR = area of region of interest in mm^2^; MD = mean density of region of interest; SD = standard deviation of mean density; Range = density range within region of interest.

Diagnostic investigation was initiated with angiotomography of the supra-aortic arterial and venous trunks (Figure 4), showing an oval-shaped lesion with the density of soft tissues and regular outline, situated lateral of the mid third of the right sternocleidomastoid muscle, compressing the external jugular vein medially, and measuring approximately 25 × 16 × 10 mm in diameter. The lesion had higher density than the adjacent musculature and lymph nodes, during the phase of the examination without contrast, and exhibited discrete uptake of contrast during the phase with contrast. The imaging exams and physical examination were suggestive of a number of diagnostic hypotheses: hemangioma, lymphangioma, or branchial cyst.

Since the patient’s symptom of significant localized pain was limiting, and since the esthetic impact upset her greatly, conventional surgical treatment was performed with excisional resection of the mass and proximal and distal ligature of the right external jugular vein. The operation was conducted by a multidisciplinary team, in which a head and neck surgeon participated. Surgery and the postoperative period were free from complications and the patient’s symptoms improved. She was discharged from hospital on the same day.

**Figure 5:**
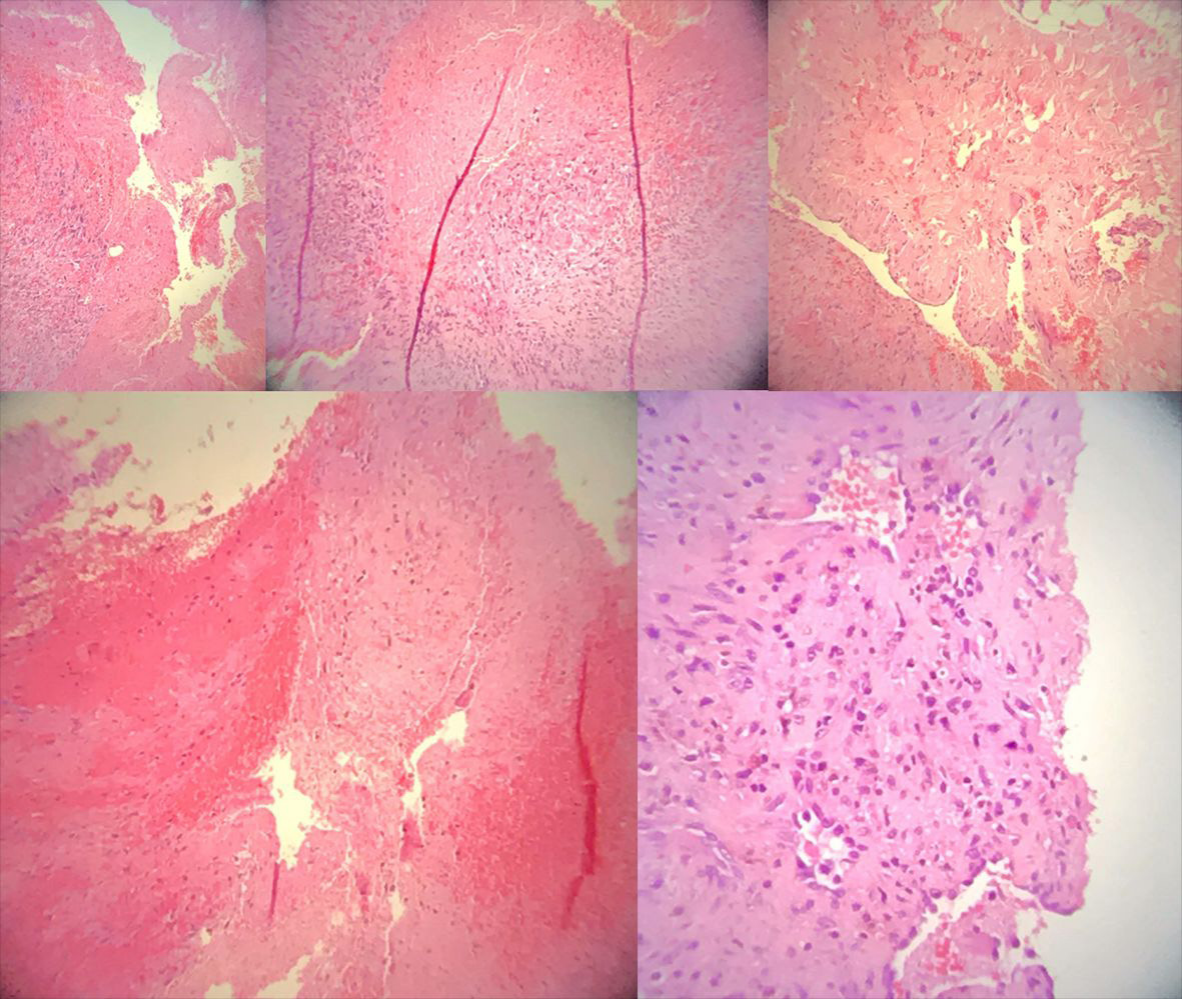
Histopathological examination by the paraffin block method. Hemangioma with thrombosis of the external jugular vein.

Definitive diagnosis was made on the basis of macroscopic anatomopathological examination of the specimen, which was an irregular, chestnut colored, elastic, lobulated tissue fragment measuring 1.5 × 1.0 × 0.5 cm, identified histopathologically as: hemangioma of the external jugular vein with thrombosis. Histological sections (Figure 5) showed fibrous connective tissue with proliferation of small vessels containing red blood cells. Also present were organized endothelial cells, fibroblasts, and fibrin forming a thrombus in the vessel interior, the lumen of which was delimited by internal elastic lamina and smooth muscle cells. The patient is still in outpatients follow-up, is satisfied with the esthetic results, and is free from pain.

## DISCUSSION

Hemangiomas are very common vascular anomalies, but are also very generic. Over the years, vascular anomalies have been the subject of much study and many different classifications and new concepts have been proposed.^1^^,^ 3 These diseases exhibit clinical manifestations and deformities that are hard to understand and, although the great majority are benign,^2^ they demand a great deal of multidisciplinary medical responsibility dedicated to workup and management of cases.

Although these tumors are common, the presentation described in this case is extremely rare, with fewer than 10 cases published in the literature of tumors originating in the wall of a vessel, and even rarer if the vessel in question is the external jugular vein.^4^^-^ 6

For many years, the term hemangioma encompassed tumors with proliferation of endothelial cells, abnormal vascular networks in tissues, and other abnormalities involving vascular dilation. Nowadays we know them as lesions in which angiogenesis is the central factor.^2^

Hemangiomas are more common among children, people with white skin, and women. In 80% of cases they are single lesions and in 20% there are multiple lesions. With regard to anatomic distribution, studies demonstrate that head and neck hemangiomas account for 60% of cases, while 25% involve the trunk and 15% the extremities.^1^^,^ 3

Conceptually, hemangiomas are caused by abnormal endothelial cell proliferation^1^^,^ 3 and are most common in childhood, during which they exhibit phases of growth, quiescence, and involution.^2^ The primary management approach is clinical watchful treatment, but in some cases total surgical excision may be the ideal choice as a definitive treatment.

Hemangiomas can originate in capillary, venous, arterial, and lymphatic tissues, with or without fistulization, but they are predominantly venous. Cellular and histopathological analysis is fundamental for definitive diagnosis.^1^^-^ 3 ^,^ 7 ^-^ 9

Imaging exams are essential to guide diagnosis and definitive treatment, and Doppler ultrasonography should be the first examination ordered at the start of work-up to investigate this pathology.^10^

Magnetic resonance imaging and angiotomography are better for assessing presentation and anatomic relationships, helping to define treatment, whether palliative (embolization of the mass to provoke regression of the mass and the symptoms it causes), or definitive (surgical excision).^10^ While angiographs are not commonly requested as a diagnostic method, they can be beneficial to diagnosis, identifying presence of microfistulas. However, in many cases the results can be normal and difficult to interpret.

Surgical treatment is indicated whenever the disease creates limitations for the patient because of esthetics and the extent to which the disease interferes with social relationships and motor activity should always be considered, as should complications, particularly when related to senses such as smell, hearing, and sight or when swallowing is affected.^7^^,^ 8 The complication most frequently involved in indications for treatment is compression of local structures, which can cause more serious symptoms, such as the thrombosis of the vessel seen in the case described here. In cases with few or no symptoms, the risk-benefit relationship of any procedure should be evaluated, aiming to impede disease progression and avert potential future complications. Treatment options include watchful waiting with clinical treatment, embolization, and surgical excision, and treatment should be chosen on a case-by-case basis according to the symptoms and limitations of each patient.

Although there is sometimes a chance of relapse, surgery to perform resection of the tumor is currently the treatment that offers the best results. This was the treatment carried out in the case described here, curing the disease and improving the patient’s symptoms.

More comprehensive data and studies of the evolution and course of this disease are still lacking in the literature, because of its rarity.

## CONCLUSIONS

Hemangioma of the external jugular vein is an extremely rare pathology, which should be suspected on the basis of physical examination findings, anatomic location, and family history. Imaging exams are fundamental for diagnosis and surgical planning.

We recommend treatment for all patients with esthetic or anatomic complications. The majority of patients are treated conservatively with watchful waiting. The surgical treatment of choice is total resection of the lesion, where possible. We believe that this is the treatment that offers the greatest certainty of cure.

Since this is a rare pathology, there is scant literature on it and more publications are needed to extend understanding and eliminate existing doubts about the pathology.
