# Supplementary material for: External jugular vein hemangioma: case report
Source: J Vasc Bras. 2019 Apr 17;18:e20180026. doi: 10.1590/1677-5449.180026 (PMC6629457; doi:10.1590/1677-5449.180026)
Supplement: Translation

# Translation: Hemangioma de veia jugular externa: relato de caso

**Authors:** Julio Cesar Peclat de Oliveira, Fernando Tebet Ramos Barreto, Bernardo de Castro Abi Ramia Chimelli, Lucas Maia Peclat de Oliveira, Ricardo Krapp Tavares, Thaysa Fernandes Lacerda da Costa, Diogo Di Battista de Abreu e Souza, Bianca Gutfilen

**Affiliations:** 1 Universidade Federal do Rio de Janeiro – UFRJ, Departamento de Radiologia, Rio de Janeiro, RJ, Brasil.; 2 Clínica Julio Peclat – CJP, Cirurgia Vascular, Rio de Janeiro, RJ, Brasil.; 3 Instituto Nacional do Câncer – INCA, Rio de Janeiro, RJ, Brasil.

**Keywords:** veia jugular externa, massa em pescoço, hemangioma

## Abstract

Hemangioma é um tumor frequente, geralmente diagnosticado em crianças, constituindo quase 10% das neoplasias benignas. Um hemangioma com crescimento na parede de um vaso é extremamente raro, e deve ser diferenciado de outras malformações vasculares de mesma origem. Apresentamos um caso raro de hemangioma de veia jugular externa e discutimos sua propedêutica e manejo.

## INTRODUÇÃO

As anomalias vasculares e tumores vasculares podem se apresentar como uma massa isolada em cabeça e pescoço em adultos. Sua classificação e subdivisão em hemangiomas e malformações vasculares sempre foi de difícil e complicado entendimento, uma vez que, com o passar dos anos e com o estudo dessas anomalias, foi proposta uma variedade de classificações e conceitos^1^^-^ 3. Malformações vasculares e hemangiomas originados na parede de um vaso, e especificamente oriundos da veia jugular externa, são extremamente raros, com menos de 10 casos publicados na literatura^4^^-^ 6. O tratamento desse tipo de condição é realizado principalmente pela estética, por tratar-se de um tumor benigno.

Apresentamos o caso de uma paciente sem antecedentes de massa ou tumor vascular, que apresentou uma ectasia venosa em região cervical, em topografia de veia jugular externa, com sintoma de dor.

## DESCRIÇÃO DO CASO

Paciente do sexo feminino de 28 anos, médica, praticante de atividades físicas regulares, sem doenças previamente conhecidas, queixava-se de massa em região cervical direita (Figura 1), dolorida à movimentação do pescoço, não sabendo informar tempo do inicio dos sintomas. A paciente não tinha história de malformação vascular na família.

**Figura 1:** Massa cervical direita.

Apresentou na primeira consulta um eco-Doppler vascular de pescoço (Figura 2), realizado em 25/11/16, que evidenciou o seguinte: veia jugular externa direita apresenta ectasia segmentar sem trombos na luz, medindo 3,6 mm no seu maior diâmetro. Foi realizado tratamento conservador, com exames periódicos e acompanhamento da massa. Porém, paciente iniciou sintoma de dor no local, acompanhada de endurecimento.

**Figura 2:** Eco-Doppler mostrando ectasia sem trombos na luz, fluxo sanguíneo normal.

Ao exame físico, notou-se abaulamento da região cervical direita, com endurecimento e leve flogose do trajeto de veia jugular externa. A paciente não apresentava outros sinais, e seu único sintoma era dor local. Sendo assim, foi medicada com rivaroxabana, pela aparente tromboflebite local, e acompanhada periodicamente com exames de imagem.

Após 1 ano de tratamento clínico, o eco-Doppler foi realizado em 14/12/17 (Figura 3), evidenciando formação nodular hipoecogênica heterogênea cervical alta à direita medindo cerca de 2,4 × 1,5 × 0,9 cm na topografia da veia jugular externa direita, com sinais de trombose na veia jugular externa. A paciente apresentou pouca melhora dos sintomas apresentados na primeira consulta, mesmo após o tratamento clínico com rivaroxabana.

**Figura 3:** Eco-Doppler mostrando massa hipoecogênica e veia jugular externa trombosada, sem fluxo.

Prosseguiu-se a investigação com angiotomografia de troncos supra-aórticos arteriais e venosos (Figura 4), a qual evidenciou lesão ovalada com densidade de partes moles e contornos regulares, situada lateralmente ao terço médio do músculo esternocleidomastoideo direito, comprimindo medialmente a veia jugular externa e medindo cerca de 25 × 16 × 10 mm de diâmetro. A lesão tinha densidade mais alta que a musculatura adjacente e linfonodos na fase sem contraste e captava discretamente o contraste na fase com contraste. Os exames de imagem e exame físico levantaram algumas hipóteses diagnósticas: hemangioma, linfangioma e cisto branquial.

**Figura 4:** Reconstruções de angiotomografia demonstrando a massa tumoral e sua relação com as estruturas vasculares do pescoço. CDPI, Barra da Tijuca, RJ. AR = área da região de interesse em mm^2^; MÉD = densidade média da região de interesse; PAD = desvio padrão da densidade média; Intervalo = intervalo de densidade dentro da região de interesse.

Como o sintoma apresentado, a dor local importante, constituía uma limitação à paciente, e esteticamente a incomodava demais, foi indicado tratamento cirúrgico convencional com ressecção excisional da massa e ligadura proximal e distal da veia jugular externa direita, o qual foi realizado de forma multidisciplinar, em conjunto com um cirurgião de cabeça e pescoço. A cirurgia e o pós-operatório transcorreram sem complicações, com melhora dos sintomas. A alta hospitalar ocorreu no mesmo dia.

O diagnóstico definitivo, obtido através da análise do exame anatomopatológico macroscópica da peça, foi de fragmento irregular de tecido com 1,5 × 1,0 × 0,5 cm, acastanhado, elástico e lobulado, tendo como conclusão histopatológica: hemangioma de veia jugular externa com trombose. Os cortes histológicos (Figura 5), mostraram tecido fibroconjuntivo com proliferação de pequenos vasos contendo hemácias. Foi constatada a presença de células endoteliais, fibroblastos e fibrina organizados formando trombo no interior do vaso, cujo lúmen era delimitado pela lâmina elástica interna e células musculares lisas. A paciente continua em acompanhamento ambulatorial e encontra-se satisfeita com resultado estético e sem dor.

**Figura 5:** Exame anatomopatológico pelo método de inclusão em parafina. Hemangioma com trombose da veia jugular externa.

## DISCUSSÃO

O hemangioma é uma anomalia vascular muito comum, e ao mesmo tempo muito genérica. Ao longo dos anos estudaram-se muito as anomalias vasculares, e foram propostas diversas classificações e novos conceitos^1^^,^ 3. São doenças que apresentam manifestações clínicas e deformidades de difícil compreensão, apesar de benignas na sua grande maioria^2^, exigindo assim grande responsabilidade médica multidisciplinar na propedêutica e na conduta dos casos.

Apesar de serem tumores comuns, sua apresentação como vista no caso é raríssima, havendo menos de 10 casos publicados na literatura de tumores oriundos da parede de um vaso, e ainda mais rara quando falamos especificamente da veia jugular externa^4^^-^ 6.

O hemangioma é um termo que durante muitos anos abrangeu tanto tumores com proliferação de células endoteliais quanto redes vasculares anormais nos tecidos e até mesmo outras alterações com dilatação vascular. Sabemos hoje em dia que são lesões em que a angiogênese é o fator central^2^.

Hemangiomas são mais comuns em crianças, indivíduos de cor branca e mulheres. São únicos em 80% dos casos e múltiplos em 20%. Com relação à distribuição anatômica, estudos demonstram que hemangiomas de cabeça e pescoço representam 60% dos casos; de tronco, 25%; e de extremidades, 15%^1^^,^ 3.

Conceitualmente, os hemangiomas são determinados por uma proliferação anormal celular do endotélio^1^^,^ 3, apresentando-se mais comumente na infância em fases evolutiva, estacionária e involutiva^2^. Sua principal forma de manejo é o tratamento clínico expectante, mas em alguns casos a excisão total cirúrgica pode ser a escolha ideal como tratamento definitivo.

Além disso, podem ser oriundos de uma combinação de tecidos capilares, venosos, arteriais e linfáticos, com fistulização ou não, mas são predominantemente venosos. A análise celular e histopatológica é fundamental no diagnóstico definitivo^1^^-^ 3 ^,^ 7 ^-^ 9.

Os exames de imagem são essenciais para direcionamento diagnóstico e condução do tratamento definitivo, sendo o eco-Doppler o primeiro a ser solicitado na investigação da patologia^10^.

A ressonância nuclear magnética e a angiotomografia demonstram melhor apresentação e relação anatômica, ajudando na definição do tratamento, seja ele paliativo (embolização da massa para regressão da massa e dos sintomas), ou definitivo (excisão cirúrgica)^10^. A realização de angiografias como método diagnóstico, apesar de infrequente, pode apresentar benefícios ao diagnóstico, identificando a presença de microfístulas. Entretanto, em muitos casos o exame pode ser normal e de difícil interpretação.

O tratamento cirúrgico está indicado sempre que a doença é limitante para o paciente devido à estética, mas deve-se sempre questionar o quanto a doença interfere na relação social e na atividade motora, ou devido a complicações, principalmente quando estas estão relacionadas aos sentidos como olfato, audição, visão e deglutição^7^^,^ 8. A complicação mais frequentemente implicada na indicação do tratamento é a compressão de estruturas locais, podendo levar a sintomas mais relevantes como a trombose do vaso no caso apresentado. Em casos com poucos ou nenhum sintoma, cabe avaliar o risco/benefício da realização do procedimento, visando impedir a progressão da doença e uma potencial complicação futura. As opções terapêuticas incluem tratamento clínico expectante, embolização e excisão cirúrgica, e o tratamento deve ser escolhido individualizando os sintomas e as limitações de cada paciente.

Apesar de por vezes ainda haver chance de recidiva, a cirurgia para ressecção do tumor é o tratamento atual com os melhores resultados. Foi o tratamento realizado no caso descrito, levando à cura da doença e à melhora dos sintomas da paciente.

Maiores dados e estudos sobre a evolução e a progressão da doença ainda são escassos na literatura, devido à sua raridade.

## CONCLUSÃO

O hemangioma de veia jugular externa é uma patologia extremamente rara, que deve ser suspeitada no exame físico, levando-se em consideração localização anatômica e história familiar. Os exames de imagem são fundamentais ao diagnóstico, e programação cirúrgica.

Indicamos o tratamento a todos os pacientes com complicações estéticas ou anatômicas. Pacientes em sua maioria são tratados de forma conservadora e expectante. A terapia cirúrgica de escolha é ressecção total da lesão, se possível. Entendemos que essa é a terapia que confere maior certeza de cura.

Por se tratar de patologia rara, a literatura é escassa, e maiores publicações são importantes para melhor entendimento e para eliminar dúvidas existentes acerca da patologia.
